# Supplementary material for: The Impact of a Low-Carbohydrate Diet on Micronutrient Intake and Status in Adolescents with Type 1 Diabetes
Source: Nutrients. 2023 Mar 15;15(6):1418. doi: 10.3390/nu15061418 (PMC10051868; doi:10.3390/nu15061418)
Supplement: Supplementary file 1 [file nutrients-15-01418-s001.zip › nutrients-2247586-supplementary.pdf]

Supplementary File

This File has been provided by the authors to give readers additional information about their work. Supplement to: Neriya Levran, Orit Pinhas-Hamiel, et al.

File S1:

Cooking workshop

The workshop's objectives were to explain the benefits of a low-carbohydrate diet and to promote skills in assembling and preparing low-carbohydrate dishes.

The syllabus of the workshop - 8 hours

Part 1: A lecture was given by a dietician regarding the aims of the study, the rationale of the low-carbohydrate diet and its apparent advantages, insulin administration while on such diet, and general information regarding preferred types of foods and daily diet managing.

Part 2: Practical section. The participants prepared about 13 different dishes and became familiar with the main food products that are used in a low-carbohydrate cuisine. During this phase, the participants consumed the foods they prepared and administered insulin, with the guidance of a dietician.

At the end of the cooking workshop, each participant received a booklet with recipes s according to the low-carbohydrate diet, a shopping list and a detailed weekly menu that lists the amount of carbohydrates for particular meals and foods.
Participants were instructed to reduce by 10% their basal insulin after initiating the LCD.

Individual meetings with the dietitian

Each participant met individually, for a 60-minute session, with the dietician in weeks 1,2,4,7,10,12, and 24 of the intervention. Dietary education sessions started with weight and waist circumference measures. After blood pressure and ketone values were measured, the dietician uploaded the glycemic, insulin and carbohydrate data from the pump, CGM and food manager applications (24-hour recall). The dietician asked for feedback, led a discussion on problem-solving, and set clear objectives based on glycemic control and upcoming events (holidays, etc.). At the end of each meeting, a detailed menu was distributed, with recipes and more examples of daily menus.

A WhatsApp group was managed by the dietician, who added diet recipes, notes (prior to holidays) and notifications about upcoming measurements and electronic questionnaires, and reminders for visits.

At the end of the study, after the data analysis, the participants were provided all of their individual data and calculations of trends and normal range values of the nutritional and health parameters that were assessed during the 24-week intervention.

The low carbohydrate diet
The daily amount of carbohydrates should be about 50-80 grams. There was no caloric restriction. |
The following foods should be avoided: milk, grains, pasta, bread, legumes, fruits and vegetables high in sugar, sugar, and honey.

Below are examples of a two-day menu:

	Day 1	Carbohydrate (gram)	Day 2	Carbohydrate (gram)
Breakfast	Cheese omelette	**8**	2 slices of low carbohydrate bread+ 40 gr of cheese 9%+	**14**

			1 cucumber+ 1/2 medium tomato+ 5 ml olive oil	
Lunch	Seasoned chicken breast with 2 cups of cauliflower (150 gr) and broccoli (112 gr) in the oven	**15**	3-4 chicken patty (40 gr each) + 1/5 cup of cauliflower rice (200 gr)	**18**
Dinner	Tuna pizza (305 gr)	**10**	Salmon in pesto +salad: Lettuce (55gr) +medium tomato + cucumber + 1/2 green pepper	**20**

Snacks:

Between the three main meals, two small meals can be eaten during the day.
The following list contains ideas for snacks, and the number of carbohydrates of the items.
130 ml of yogurt, 5.5 g
A small apple, 15 g
10 walnuts, 6 g
25 gr of dark chocolate 85% coca 4.4 gr

Examples of items for a shopping list.
Eggs
Fish
Meat
Chicken
Vegetables: cabbage, cauliflower, broccoli, cucumber, lettuce, brussels sprouts, zucchini, spinach, green beans, avocado

In limited quantities: tomato, red pepper, green pepper, carrot**.**
Dairy products: cheeses-mozzarella, parmesan, cheddar, mascarpone, goat cheeses**.**
Milk substitutes: almond milk, soymilk, cream 38%, coconut water.
Flours: almond flour, ground flax seeds, coconut flour**.**
Chocolate: 85%-90% cocoa solids
Almond butter, peanut butter, hazelnut butter, milk butter, chia**.**
Olive oil
weeteners: Sweetango, liquid stevia, monk fruit, Erythritol, Laknanto**.**

Below are the main topics that we focused on during the eight sessions.

Session 1:
Adjusting insulin and the menu more precisely to participants' preferences, and in accordance with the research guidelines

Session 2:
Special concerns, according to the patients' requests: physical activity, bowel movements, hypoglycemia, etc.

Session 3:
Maintaining a low-carb diet when eating out, and at family and social events, holidays, and parties.

Session 4:
Healthy eating and eating habits, including avoidance of processed food, avoidance of screen time while eating, and family meals.

Session 5:
Lipids and fats, their proportions in various food items, and the effect on metabolic health

Session 6: Motivational session

Session 7: Conclusion

Each participant had the option to contact the caregiver and dietician any time during the intervention.
Two motivational phone reach outs were conducted by the dietician.
The participants continued regular visits with their endocrinologists.
